# Relationship between Persistent Gastrointestinal Symptoms and Duodenal Histological Findings after Adequate Gluten-Free Diet: A Gray Area of Celiac Disease Management in Adult Patients

**DOI:** 10.3390/nu13020600

**Published:** 2021-02-12

**Authors:** Gloria Galli, Marilia Carabotti, Emanuela Pilozzi, Edith Lahner, Bruno Annibale, Laura Conti

**Affiliations:** 1 Medical-Surgical Department of Clinical Sciences and Translational Medicine, Sant’Andrea Hospital, Sapienza University of Rome, 00189 Roma, Italy; Marilia.carabotti@uniroma1.it (M.C.); edith.lahner@uniroma1.it (E.L.); bruno.annibale@uniroma1.it (B.A.); l.conti@uniroma1.it (L.C.); 2 Department of Clinical and Molecular Medicine, Sant’Andrea Hospital, Sapienza University of Rome, 00185 Roma, Italy; emanuela.pilozzi@uniroma1.it

**Keywords:** celiac disease, gluten-free diet, gastrointestinal symptoms, malabsorption signs, duodenal histology, slow responders

## Abstract

A gluten-free diet (GFD) leads to a rapid improvement in gastrointestinal (GI) symptoms, biochemical alterations and duodenal histological damage in the majority of celiac disease (CD) patients. This study aimed to assess the frequency and factors associated with the persistence of GI symptoms/malabsorption signs and their relationship with duodenal histological findings among CD patients on an adequate GFD (mean duration 16 months, range 12–28 months). This longitudinal cohort study included 102 adult CD patients (median age 38.5 years, range 18–76 years, F = 71.6%) diagnosed between 2012 and 2018. A total of 36.3% of the included patients had persistent GI symptoms and/or malabsorption signs (Group 1), while the remaining patients had complete GI well-being without malabsorption signs (Group 2) at the time of histological re-evaluation. The persistence of GI symptoms/signs was associated with a long duration of symptoms/signs before CD diagnosis (≥5 years) (OR 5.3; 95% CI 1.3–21.8) and the presence of constipation at the time of CD diagnosis (OR 7.5; 95% CI 1.3–42) while for other variables, including age at CD diagnosis, sex, duration of GFD, comorbidities, CD serology positivity and severity of duodenal damage at histological re-evaluation, no association was found. According to our results, the persistence of symptoms/signs is not associated with histological findings, and their relationship could be a gray area in CD management.

## 1. Introduction

Celiac disease (CD) is an immune-mediated enteropathy affecting approximately 1% of the Western population [1]. CD can present with highly variable clinical manifestations, often characterized by nonspecific and subtle symptoms frequently leading to delayed diagnosis, making it a clinical challenge for physicians. The only available therapy is a life-long strict gluten-free diet (GFD). The main goal of the GFD is the amelioration/disappearance of symptoms and biochemical alterations reported at CD diagnosis, as well as histological healing and the avoidance of CD-related complications [2,3,4]. Since an inadequate GFD has been reported to be the most frequent factor associated with gastrointestinal (GI) symptom persistence [5,6], adherence should be assessed at each follow-up visit [1,2]. Beginning a GFD can promptly improve GI symptoms [7], but a percentage of CD patients, ranging from 7 to 30%, may complain of persistent symptoms [8,9]. Although these patients could be labeled as having nonresponsive CD (NRCD) [10], the most recent European Guidelines discouraged the use of this definition, suggesting the term “slow responders”, since most of them will improve over time on a strict GFD [1]. Regarding malabsorption signs, only a few studies have described their persistence after the start of a GFD [11]. Furthermore, it has been demonstrated that a substantial percentage (ranging from 9% to 43%) of celiac patients do not achieve complete mucosal healing after the start of the GFD [4,12,13]. Conflicting data on histological recovery are mainly dependent on the variability of GFD duration and different GFD adherence assessments used at the time of histological control in different studies [14,15,16,17]. Refractory celiac disease (RCD), a rare complication of CD, is defined as the persistence or recurrence of GI symptoms and signs of malabsorption with persistent villous atrophy despite a strict GFD for more than 12 months when other potentially accountable disorders are excluded [1,18].

The role of duodenal biopsies is essential to distinguish between RCD and CD patients with slow clinical recovery, even if it is not the only decisive tool. Nevertheless, the usefulness and correct timing of routine follow-up esophagogastroduodenoscopy (EGDS) with intestinal biopsies in celiac patients on a GFD are currently under debate. Some experts have proposed performing it only in selected patients [19]; the most recent European position paper on the management of CD has suggested that it seems reasonable to perform it 1–2 years after the beginning of the GFD in CD adults to assess mucosal healing, especially in patients with initially severe clinical presentations or in patients with a CD diagnosis over 40 years of age [1].

This study aimed to assess the frequency and possible factors associated with the persistence of GI symptoms/malabsorption signs and their relationship with duodenal histological findings at the time of histological re-evaluation in a cohort of adult celiac patients on an adequate GFD.

## 2. Materials and Methods

**Figure 1:**
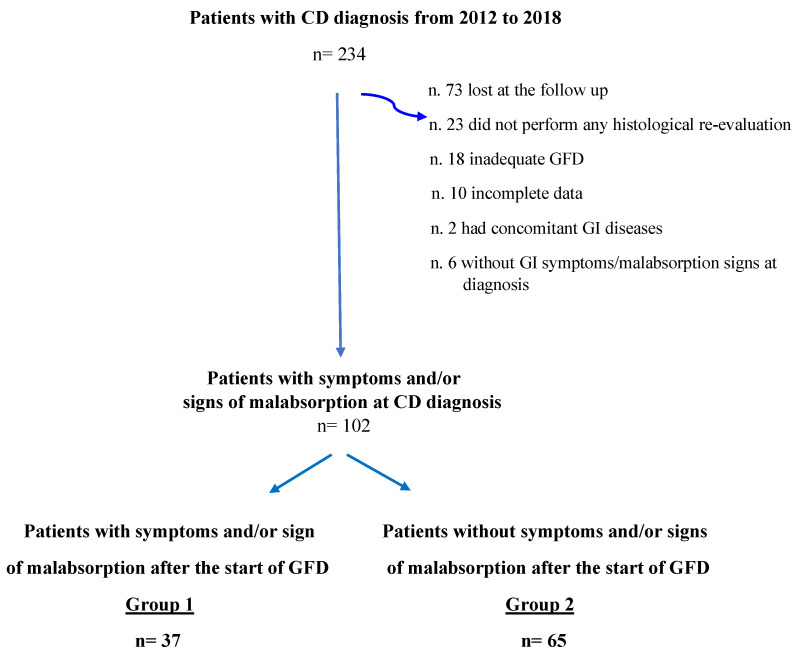
Flowchart of study population. CD = Celiac Disease; GI = Gastrointestinal; GFD = Gluten-free diet.

This is a longitudinal cohort study focusing on CD patients diagnosed and followed up between 2012 and 2018 at our academic tertiary referral center (Sant’Andrea University Hospital of Rome). For the purpose of this study, patients were included when they met the following inclusion criteria: (1) adult patients (≥18 years old); (2) CD diagnosis based on positive CD-specific serology (anti-transglutaminase IgA and anti-endomysium IgA autoantibodies, normal total IgA) and concomitant duodenal villous atrophy classified as Marsh 3A-C damage [20]; (3) GI symptoms and/or signs of malabsorption at the time of CD diagnosis; (4) histological re-evaluation performed in a period ranging from 12 to 28 months after the beginning of a GFD; (5) an adequate GFD, assessed by Biagi score [21] at the time of histological re-evaluation (Biagi score ≥ 3); and (6) complete structured questionnaire comprising personal and clinical data including concomitant diseases and drugs taken at diagnosis and follow-up visits. Patients with other concomitant relevant GI diseases (i.e., inflammatory bowel diseases, intestinal parasitosis, sartan enteropathy, and common variable immunodeficiency) were excluded from the study (Figure 1). The time span between the GI symptoms/malabsorption signs leading to the diagnosis and the diagnosis of CD was assessed and reported at the first visit.

All data from the included CD patients were anonymized to guarantee the secure processing of sensitive data and collected into a predefined spreadsheet. The study was conducted according to the Sapienza Sant’Andrea Hospital protocol, and written informed consent was obtained from all included patients at the time of CD diagnosis. The study protocol conforms to the ethical guidelines of the 1975 Declaration of Helsinki, as reflected in a priori approval by the institution’s human research committee.

### 2.1. Endoscopic Procedures and Histological Classification

EGDS with at least four biopsies obtained from the second part of the duodenum was performed in all patients using a flexible video-gastroscope (Olympus GIF-Q165, GIFQ185). All included patients underwent EGDS at diagnosis and histological re-evaluation in a period ranging from 12 to 28 months after beginning an adequate GFD.

The same expert pathologist in the field of CD (E.P.), who was blinded to clinical data, examined the intestinal biopsies for each patient both at diagnosis and follow-up. Biopsies were analyzed after hematoxylin and eosin and immunohistochemical staining for CD3 counts and were assessed using the Marsh classification system modified by Oberhuber [20,22]. The histological persistence of the above-listed alterations was described and classified as Marsh 1, Marsh 2 or Marsh 3 (A, B or C), as previously defined. To assess the concomitant presence of *Helicobacter pylori* (*H.p*.) infection and/or other gastric diseases, at least five gastric biopsies (1 from the lesser and 1 from the greater curve of the antrum, 1 from angular incisura and 2 from the corpus/fundus) were taken at CD diagnosis and at the time of re-evaluation according to the updated Sydney system [23].

### 2.2. Serological Assays

Anti-transglutaminase (tTG) and anti-endomysium (EMA) IgA antibodies were assessed in all patients both at diagnosis and at the time of histological re-evaluation. IgA tTG antibodies were assayed using an enzyme-linked immunosorbent assay kit commercially available from Eurospital (Trieste, Italy). An indirect immunofluorescence assay was used to detect IgA EMA in monkey esophageal sections. Other blood assays, such as complete blood cell count, ferritin, folate, vitamin B12, total cholesterol, triglycerides, total protein count and albumin, were performed using standard laboratory techniques to investigate the presence of associated signs of malabsorption. Values of biochemical alterations were taken into consideration according to their standard laboratory ranges.

### 2.3. GI Symptoms and GFD Assessment

The presence, frequency and intensity of GI symptoms were assessed at diagnosis and at the time of histological re-evaluation through a standardized questionnaire currently used in our department [24], including the Bristol scale [25].

Upper GI symptoms, such as vomiting/nausea, heartburn, regurgitation, dysphagia and troublesome postprandial fullness/early satiety, were considered if they were present at least once a week for at least the last 3 months [26]. Lower GI symptoms, abdominal pain and troublesome abdominal bloating were considered if they were present with at least a weekly frequency; constipation was defined as fewer than 3 spontaneous bowel movements per week or straining, with lumpy hard stools (Bristol scale 1–2); and diarrhea was defined as increased frequency (>3 stools/day) or decreased consistency (loose or liquid stools, Bristol scale 6–7) of bowel movements for at least 3 months before the CD diagnosis [27]. Included patients were compared and divided into two groups on the basis of persistence (Group 1) or complete resolution (Group 2) of GI symptoms and/or malabsorption signs at the time of histological re-evaluation. GI symptoms were compared before and after the start of the GFD to evaluate possible clinical changes after a GFD, resulting in three different clinical pictures: disappearance (when totally regressed), amelioration (when improved in intensity and/or frequency), persistence (when remaining stable or worsened in intensity and frequency) and new onset (if newly appeared).

GFD compliance was assessed by the five-point validated Biagi score [21], consisting of four questions about how patients managed their GFD (0–2 = voluntary gluten ingestion, not adequate GFD; 3–4 = adequate GFD); this score was administered by two dedicated physicians during follow-up visits. Patients were also instructed and specifically interviewed by the two dedicated physicians to rule out gluten occult contaminations.

### 2.4. Statistical Analyses

Descriptive statistics are expressed as numbers, percentages (%) of totals and medians (ranges). Univariate analyses were performed by *t*-test, Fisher’s exact test and/or by chi-squared test for continuous or categorical variables to identify differences between CD patients with or without persistent symptoms/signs at the time of histological re-evaluation. Odds ratios (ORs) and 95% confidence intervals (CIs) were used to identify variables related to the dependent variable of interest (persistence of symptoms/signs at histological re-evaluation) and were obtained by logistic regression analysis. Age at CD diagnosis (>40 years), sex, duration of diagnostic symptoms and/or signs before CD diagnosis (>5 years), duration of the GFD (more than 18 months, range 19–28 months), associated autoimmune diseases, presence of constipation at CD diagnosis, persistence of specific antibody positivity after the GFD, presence of Marsh 3C duodenal damage and *H.p.* infection positivity at the time of histological re-evaluation were included in the logistic model. Two-tailed *p* values < 0.05 were considered statistically significant. Statistical analyses were performed by MedCalc© Statistical (MedCalc Software bv, Ostend, Belgium).

## 3. Results

A total of 234 patients with a new diagnosis of CD made between 2012 and 2018 at our referral center for CD were eligible for the study. Of these patients, a total of 132 (56.4%) were excluded because they did not meet the inclusion criteria (Figure 1). Finally, 102 patients (median age 38.5, range 18–76, female sex 71.6%) presenting GI symptoms and/or signs of malabsorption at the time of CD diagnosis were included in the study. Specifically, 23 (22.5%) patients presented only with GI symptoms, 10 (9.8%) patients presented only malabsorption signs and 69 (69.2%) patients reported both GI symptoms and malabsorption signs. A total of 37 (36.3%) patients had persistent GI symptoms and/or malabsorption signs (Group 1), while 65 (63.7%) stated complete GI well-being in the absence of malabsorption signs (Group 2) at the histological and clinical follow-up performed after a median period of 16 months (range 12–28) of an adequate GFD. Table 1 shows the main demographic, clinical, biochemical and histological characteristics of Group 1 and Group 2 at the time of CD diagnosis. No significant differences were found between the two groups concerning sex, body mass index, family history of CD, autoimmune or other relevant (cardiovascular and metabolic) comorbidities. Specifically, four patients in Group 1 and eight in Group 2 had concomitant autoimmune thyroiditis and had euthyroidism at the time of CD diagnosis. Celiac patients with persistent GI symptoms and/or malabsorption signs (Group 1) at the time of histological re-evaluation presented a longer duration of diagnostic symptoms and/or signs before CD diagnosis (≥5 years) than Group 2 (*p* = 0.04). Regarding *H.p.* infection, 27% and 26.1% of Groups 1 and 2, respectively, presented and received eradication treatment for the infection at the time of CD diagnosis. Concerning clinical presentation at CD diagnosis, 64.8% of Group 1 and 69.2% of Group 2 presented with both GI symptoms and malabsorption signs, without any significant differences between the two groups. Considering patients presenting with only malabsorption signs or GI symptoms at CD diagnosis, no significant differences were found between Group 1 (13.5% and 21.6%) and Group 2 (7.7% and 23.1%). Among the GI symptoms complained of at the time of CD diagnosis, constipation was more common in Group 1 than in Group 2 (*p* = 0.051), but without a significant difference. No significant differences were found with regard to other analyzed GI symptoms. Signs of malabsorption were similar between the two groups. In particular, anemia was present in 45.7% and 46.8% of patients, iron deficiency in 61.1% and 64.9%, folic acid and/or vitamin B12 deficiency in 34.5% and 31.9% of patients, parathyroid hormone (PTH) increase in 20.1% and 28% of patients, hypocholesterolemia in 12.1% and 8.6% of patients and hypoproteinemia in 3.4% and 4% of patients in Group 1 and Group 2, respectively. The proportion of patients with the most severe duodenal histological damage (Marsh 3C) was also similar in the two groups.

Table 2 reports the main analyzed features of CD patients at the time of histological re-evaluation. The median duration of adequate GFD before the histological re-evaluation was not significantly different between Group 1 and Group 2 (14 vs. 18 months, range 12–28, respectively, *p* = 0.2). The percentages of patients with persistent CD-specific antibody positivity and *H.p.* infection were similar in the two groups, without any significant difference.

**Figure 2:**
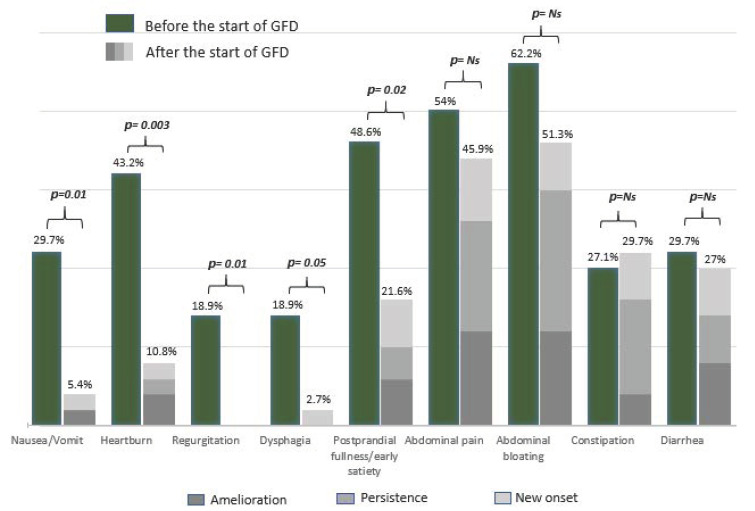
Trend of GI symptoms among patients with persistent GI symptoms/signs at the time of the histological re-evaluation. GFD = Gluten-free diet; Ns = Not significant.

Concerning complete histological recovery (Marsh 0) and persistent duodenal atrophy (Marsh 3), no differences were found between the two groups. Marsh 1 lesions were more frequent but not significantly different in patients without persistent symptoms/signs than in patients in Group 2. The frequency of each symptom at CD diagnosis and its clinical outcome (amelioration, persistence, new onset) after the beginning of the GFD are reported in Figure 2. As shown in Figure 2, abdominal bloating was the most frequent symptom reported before the beginning of the GFD (62.2%), followed by abdominal pain (54%) and dyspepsia (48.6%). After the beginning of the GFD, abdominal bloating and abdominal pain remained the most frequent symptoms (51.3% and 45.9%, respectively), followed by constipation (29.7%). Overall, upper GI symptoms significantly decreased, whereas lower GI symptoms remained almost unchanged after beginning a GFD.

As shown in Table 3, when comparing patients with (Marsh 3; *n* = 10) or without (Marsh 0–1; *n* = 27) persistence of duodenal atrophy at the time of histological revaluation, no differences were found between the two groups in terms of duration of the GFD, persistence of CD-specific serology, type of GI symptoms reported or number of patients with malabsorption signs. Among patients with persistent duodenal atrophy, two patients were finally classified as having RCD.

Logistic regression analysis (Table 4) showed that patients with a long duration of symptoms/signs (≥5 years) before CD diagnosis had a 5.3-fold increased risk (95% CI 1.3 to 21.8) of having persistent symptoms/signs at the time of histological re-evaluation. Furthermore, the proportions of patients with constipation at CD diagnosis presented a higher risk of persistent symptoms/signs at histological follow-up than patients with other types of GI symptoms at CD diagnosis (OR 7.5, 95% CI 1.3 to 42). Conversely, of other considered variables, such as sex, age at CD diagnosis, autoimmune comorbidities, duration of GFD before histological control, severity of histological damage at CD diagnosis (Marsh 3C), CD-specific serology positivity and presence of *H.p.* infection at histological re-evaluation, no significant associations with the dependent variable (persistence of GI symptoms/malabsorption signs at histological control) were found.

## 4. Discussion

A GFD leads to a rapid improvement in GI symptoms and biochemical alterations in the majority of CD patients, as reported in several studies [2,3,4,7]. In our study, more than one-third (36.3%) of CD patients presenting with GI symptoms and/or malabsorption signs at CD diagnosis had persistence of them at the time of histological re-evaluation despite an adequate GFD. The frequency of GI symptom/sign persistence found in our study was slightly higher than that in most previous papers (7% to 30%) [8,9]. Excluding the subgroup of CD patients with only the persistence of malabsorption signs without any GI symptoms (*n* = 10 patients), the above percentage consequently decreased from 36.3% to 26.5%. As shown in several papers, the most common factor associated with the persistence of symptoms/signs in CD patients is voluntary or accidental gluten ingestion [5,28]. Patients with an inadequate GFD were excluded from our study (Figure 1) by performing a validated dietary questionnaire [21]. Considering the well-known limits of traditional questionnaires for GFD adherence [1], new methods, such as fecal/urinary gluten peptide assessment, have recently been developed [29]. In our study, we tried to improve the reliability of the GFD adherence assessment by a careful interview performed by two dedicated physicians. It is also important to highlight that occasional GFD transgressions did not always influence the development of villous atrophy independently of symptoms, as shown by a recent study [30]. In addition, considering data that could indirectly provide us with information on possible gluten ingestion, such as persistent CD-specific antibody positivity and duodenal histological damage [31], at the time of histological re-evaluation, no significant differences were found between the two groups. Consequently, we may exclude inadequate GFD as an important factor associated with the persistence of GI symptoms/signs in our study population.

In fact, patients with persistent symptoms/signs did not present any more severe duodenal histological damage than the group without symptoms/signs (Table 2). Conversely, patients with persistent atrophic duodenal histological damage (Marsh 3) were frequently asymptomatic (Group 2). Few studies aiming to assess the association between mucosal healing and persistent symptoms after the GFD showed that symptoms were poorly predictive of histological duodenal atrophy persistence, confirming our results [11,17]. In contrast, a study showed a significant association between diarrhea, anemia, weight loss, heartburn, diffuse abdominal pain and constipation with persistent villous atrophy [32]. These conflicting results could be associated with the widely different timing of endoscopic/histological control (from 1 to > 8 years after the beginning of GFD) and the frequent lack of a validated GFD assessment considered in different previously published papers [11,32]. Of 10 patients with both persistence of atrophy and GI symptoms/malabsorption signs, only two patients received an RCD diagnosis. The remaining eight patients had complete histological recovery within a period of 1 to 3 additional years. This trend might suggest that some CD patients could have a “slow histological recovery”, needing many years to achieve histological healing. Therefore, we think that this is a gray area of CD management and that these patients should not be defined at once as refractory to the diet. In relation to this point, a new score for CD histological reassessment, after the beginning of the GFD, has been developed, representing a useful tool to detect an improvement in duodenal mucosa damage [33].

Our study also shows that the persistence of symptoms/signs is increased in celiac patients with a long duration of GI symptoms/signs before CD diagnosis (≥5 years) (Table 4). However, conflicting data on the delay in CD diagnosis (range: 17 months to 11 years) are present in the literature [34,35,36]. Diagnostic delay frequently occurs in CD, but its clinical consequences are still under debate. In a recent questionnaire-based study aiming to assess whether diagnostic delay influenced the improvement of GI symptoms after the beginning of a GFD, a diagnostic delay of more than 3 years was associated with a slow improvement of symptoms in CD patients on a GFD, confirming our result [34]. Among GI symptoms, the upper GI symptoms were those with a greater improvement, while intestinal movement alterations (both diarrhea and constipation) or abdominal pain/bloating (Figure 2) often showed persistence or new onset. Interestingly, the presence of constipation at CD diagnosis was the only variable significantly associated with persistent symptoms/signs at histological re-evaluation (Table 4). In our study, constipation showed a slight improvement with a notable rate of new onset at the time of histological re-evaluation (Figure 2). Even if constipation has been widely considered to be a nontypical presentation symptom at the time of CD diagnosis [37], only a few studies have reported data on its persistence/amelioration in adult patients after the beginning of a GFD [6,32]. On the one hand, the persistence/new onset of constipation could be associated with dietary changes brought on from the beginning of a GFD and a GFD might be responsible for intestinal microbiota modification; on the other hand, constipation may be a pre-existing and constitutional condition [38,39]. According to our data, we could therefore speculate that a GFD did not satisfactorily improve constipation, leading in some cases to exacerbate this symptom in CD patients. Diarrhea remained a relatively common (considering both persistence and new onset) symptom at the time of the follow-up visit, even if not significantly more common at the time of CD diagnosis in Group 1 and independent of histological recovery (Table 2 and Table 3). These data are in line with a previous study reporting a significant rate of diarrhea in patients on a short-term GFD (1–2 years) [40]. We hypothesize that the high rate of persistent diarrhea in the first years after the beginning of a GFD, as reported in our study, might also be due to dietary modification. In fact, a GFD may often lead to an increase in fiber intake with a consequent enrichment in fermentable oligosaccharides, disaccharides, monosaccharides and polyols (FODMAPs) [41]. FODMAPs are fermentable, poorly absorbed and osmotically active short molecules that are potentially responsible for GI symptoms and the alteration of intestinal microbiota in predisposed patients [38,39,41]. Even though the majority of CD patients had a low intake of gluten-free cereals and sweets, they still consumed a significant amount of vegetables and fruits high in FODMAPs, as demonstrated by a recently published study [42].

Furthermore, abdominal bloating and abdominal pain resulted in the most frequent symptoms reported in our study population before the beginning of the GFD as well as at the time of histological re-evaluation. The combination of these symptoms (constipation, diarrhea, abdominal pain and abdominal bloating) could be secondary to persistent organic intestinal damage or part of a functional disorder such as irritable bowel syndrome (IBS). IBS often overlaps with CD diagnosis in a percentage ranging from 22% to 38% of CD patients [43,44]. In this study, however, we did not systematically evaluate the occurrence of this syndrome through a standardized questionnaire since it fell outside our aims. To distinguish patients with functional disorders from patients with persistent active CD despite an adequate GFD for at least 12 months, endoscopic/histological re-evaluation is mandatory. As mentioned above, our data demonstrated the prompt amelioration of the upper GI symptoms (comprehensive dysphagia and nausea/vomiting) regardless of histological healing. In particular, dyspeptic symptoms (both postprandial fullness and early satiety) significantly improved after the beginning of the GFD. This result could be explained by the improvement of neuroimmunomodulatory alterations of the GI tract linked to duodenal histological healing owing to the start of a GFD [45]. Nevertheless, more recent studies have demonstrated the role of mast cells in GI functional disorders, such as IBS or functional dyspepsia, and the lack of their reduction after the beginning of a GFD [46]. In addition, some studies reported histological changes in the celiac stomach potentially responsible for the important prevalence of dyspepsia in CD patients at the time of diagnosis [46,47,48,49]. Although *H.p.* is a relatively common cause of dyspeptic symptoms, it was not significantly associated with the persistence of GI symptoms/signs in the logistic regression analysis.

The major strength of this study is the well-detailed inclusion criteria and consequently the well-defined cohort of celiac patients analyzed. In particular, histological re-evaluation was performed in each patient within a strict and well-defined period after the start of the GFD. Furthermore, we included only celiac patients with an adequate GFD assessed by a validated questionnaire even without the assessment of fecal/urinary gluten peptides due to their availability after the beginning of the study. We have tried to overcome this methodological limitation by improving the reliability of the GFD adherence assessment by a careful interview performed by two dedicated physicians. Another limitation of this study was the restricted number of patients included. This limitation is mainly dependent on the strict inclusion criteria considered.

## 5. Conclusions

Approximately 1/3 of CD patients on an adequate GFD had persistence of GI symptoms or malabsorption signs at the time of histological re-evaluation. Patients with long-lasting GI symptoms/malabsorption signs before CD diagnosis (≥5 years) had an increased risk of having persistent GI symptoms/malabsorption signs at the time of histological re-evaluation. Upper GI symptoms had a significant recovery after an adequate GFD was started, while constipation was the symptom with the least improvement. No significant associations were found between the persistence of GI symptoms/malabsorption signs and CD serology positivity or severity of duodenal damage at histological re-evaluation. According to our results, the persistence of symptoms/signs is not associated with histological findings, and their relationship is still a gray area in CD management.

## Figures and Tables

**Table 1:** Comparison of main characteristics of included patients at the time of CD diagnosis with respect to presence or absence of persistent GI symptoms/malabsorption signs.

*N* (%)	Patients with Persistent Sign/Symptoms Group 1 *n* = 37	Patients without Persistent Signs/Symptoms Group 2 *n* = 65	*p*
**Median age at diagnosis, yrs (range)**	41 (18–76)	38 (18–66)	0.245
**Female gender (%)**	28 (75.7)	45 (69.2)	0.648
Median BMI * Kg/m^2^ (range)	23.4 (17.5–31.8)	21.1 (16–30.8)	0.168
**Comorbidities**			
Autoimmune	9 (24.3)	17 (26.5)	1
Others ^#^	8 (18.8)	13 (20.3)	1
**Family history of CD ^§^**	8 (21.6)	8 (12.3)	0.261
**≥5 years of duration of symptoms/signs before CD diagnosis**	13 (39.4)	10 (18.8)	0.045
***H. pylori* infection**	10 (27%)	17 (26.1%)	1
**Clinical presentation**			
Only GI ° symptoms	8 (21.6)	15 (23.1)	0.807
Only malabsorption signs	5 (13.5)	5 (7.7)	0.498
Both symptoms and signs	24(64.8)	45 (69.2)	0.665
**GI symptoms**			
Total pts with GI symptoms	32 (86.5)	54 (83.1)	0.780
Nausea/vomiting	11 (29.7)	17 (26.1)	0.817
Heartburn	16 (43.2)	29 (44.6)	1
Regurgitation	7 (18.9)	14 (21.5)	0.804
Dysphagia	7 (18.9)	7 (10.7)	0.369
Postprandial fullness/early satiety	18 (48.6)	37 (56.9)	0.535
Abdominal pain	20 (54)	39 (60)	0.677
Abdominal bloating	23 (62.2)	48 (73.8)	0.264
Constipation	10 (27.1)	7 (10.8)	0.051
Diarrhea	11 (29.7)	16 (24.6)	0.643
Diarrhea			
**Signs of malabsorption**	29 (78.4)	49 (75.4)	0.811
**Marsh 3C at diagnosis**	17 (45.9)	39 (60)	0.215

* BMI = Body mass index; # Others = Metabolic, cardiovascular; § CD = Celiac disease; ° GI = Gastrointestinal.

**Table 2:** Comparison of clinical, serological and histological features at the time of histological re-evaluation among patients with or without persistence of GI symptoms/malabsorption signs.

*N* (%)	Patients with Persistent Sign/Symptoms Group 1 *n* = 37	Patients without Persistent Signs/Symptoms Group 2 *n* = 65	*p*
Median months of GFD *(range)	14 (12–28)	18 (12–28)	0.211
**Clinical presentation**			
Only GI ^§^ symptoms	18 (48.7)	0	na ^#^
Only malabsorption signs	10 (27)	0	na
Both symptoms and signs	9 (24.3)	0	na
**Antibody positivity**	8 (21.6)	15 (23.1)	1
**H. pylori infection**	1 (2.7)	4 (6.1)	0.650
**Marsh score**			
Marsh 0	26 (70.3)	39 (60)	0.392
Marsh 1	1 (2.7)	11 (17)	0.052
Marsh 2	0	0	na
Marsh 3A	7 (18.9)	14 (21.5)	0.804
Marsh 3B	2 (5.4)	1 (1.5)	0.290
Marsh 3C	1 (2.7)	0	na

* GFD = Gluten-free diet; § GI = Gastrointestinal; *#* na = Not applicable.

**Table 3:** Comparison of GFD duration, clinical and serological features among patients with (Marsh 3) or without (Marsh 0–1) persistence of duodenal atrophy at the time of histological re-evaluation.

*N* (%)	Pts with Marsh 3 at Histological Control *n* = 10	Pts with Marsh 0–1 at Histological Control *n* = 27	*p*
Median months of GFD * (range)	14 (12–25)	14 (12–28)	0.63
**Clinical presentation**			
Only GI § symptoms	5 (50%)	13 (48.2%)	1
Only malabsorption signs	4 (40%)	9 (33.3%)	0.715
Both symptoms and signs	1 (10%)	5 (18.5%)	0.347
**GI ^§^ symptoms**			
Nausea/vomiting	0	2 (7.4)	1
Heartburn	2 (20)	2 (7.4)	0.291
Regurgitation	0 (0)	0 (0)	1
Dysphagia	0 (0)	1 (3.7)	1
Postprandial fullness/early satiety	1 (10)	7 (25.9)	0.404
Abdominal pain	4 (40)	13 (48.1)	0.724
Abdominal bloating	5 (50)	14 (51.8)	1
Constipation	2 (20)	9 (33.3)	0.688
Diarrhea	3 (30)	7 (25.9)	1
Signs of malabsorption	6 (60)	13 (48.1)	0.714
**Antibody positivity**	2 (20)	6 (22.2)	1

* GFD = Gluten-free diet; § GI = Gastrointestinal.

**Table 4:** Variables associated with the persistence of symptoms/signs at the time of histological re-evaluation in the logistic regression analysis.

	Odds Ratio	95% CI	*p*
**Age >40 years**	1.9	0.63–5.72	0.25
**Female gender**	0.5	0.15–2.13	0.41
**≥5 years duration of symptoms/signs before CD diagnosis**	5.3	1.32–21.78	0.01
Duration of GFD * > 18 months	0.9	0.83–1.02	0.15
**Associated autoimmune diseases**	0.6	0.18–2.32	0.51
**Constipation at CD ^§^ diagnosis**	7.4	1.33–41.99	0.02
**Antibody positivity at histological re-evaluation**	1.4	0.38–5.76	0.56
**Marsh 3C at histological re-evaluation**	0.5	0.18–1.86	0.36
**H. pylori infection at histological re-evaluation**	0.1	0.01–1.85	0.14

* GFD = Gluten-free diet; § CD = Celiac disease.

## Data Availability

The data presented in this study are available on request from the corresponding author. The data are not publicly available due to ethical restrictions.
